# Dual energy window imaging for optimisation of *P*/*V* ratios in VP SPECT

**DOI:** 10.1186/s40658-021-00417-z

**Published:** 2021-10-16

**Authors:** A. G. G. Doruyter, J. L. Holness

**Affiliations:** 1 NuMeRI Node for Infection Imaging, Central Analytical Facilities, Stellenbosch University, Cape Town, South Africa; 2 Division of Nuclear Medicine, Faculty of Medicine and Health Sciences, Stellenbosch University, Cape Town, South Africa; 3 Nuclear Medicine Division, Department of Radiation Medicine, University of Cape Town, Cape Town, South Africa

**Keywords:** Ventilation, Perfusion, Pulmonary embolism, Lung SPECT, Count rate ratio

## Abstract

**Purpose:**

Ventilation–perfusion single-photon emission computed tomography (VP SPECT) plays an important role in pulmonary embolism diagnosis. Rapid results may be obtained using same-day ventilation followed by perfusion imaging, but generally requires careful attention to achieving an optimal count rate ratio (*P*/*V* ratio) of ≥ 3:1. This study investigated whether the ratio of counts simultaneously acquired in adjacent primary and Compton scatter energy windows (*E*_ratio_) on V SPECT was predictive of final normalised perfusion count rate (PCR_norm_) on P SPECT using [^99m^Tc]Tc-macroaggregated albumin (MAA), thus allowing for optimisation of *P*/*V* ratios.

**Methods:**

Same-day VP SPECT studies acquired using standard protocols in adult patients during a 2-year period (training dataset) were assessed. Studies were included provided they were acquired with correct imaging parameters, and injection site imaging and laboratory records were available for quality control and normalised count rate corrections. Extraction of DICOM information, and linear regression were performed using custom *Python* and *R* scripts. A predictive tool was developed in Microsoft Excel. This tool was then validated using a second (validation) dataset of same-day studies acquired over a subsequent 7-month period. Accuracy of the prediction tool was assessed by calculating the mean absolute percentage error (MAPE).

**Results:**

Of 643 studies performed, the scans of 342 participants (median age 30.4 years, 318 female) were included in the training dataset, the analysis of which yielded a significant regression equation (*F*(1,340) = 1057.3, *p* < 0.0001), with an adjusted *R*^2^ of 0.756 and MSE of 0.001089. A prediction tool designed for routine clinical use was developed for predicting final *P*/*V* ratio. Of an additional 285 studies, 198 were included in the second (validation) dataset (median age 29.7 years, 188 female). The Excel-based tool was shown to be 91% accurate (MAPE: 9%) in predicting *P*/*V* ratio.

**Conclusion:**

The relationship between the ratio of simultaneously acquired counts in adjacent energy windows on V SPECT and perfusion count rate after administration of a known activity of [^99m^Tc]Tc-MAA can be linearly approximated. A predictive tool based on this work may assist in optimising the dose and timing of [^99m^Tc]Tc-MAA administration in same-day studies to the benefit of patients and workflows.

**Supplementary Information:**

The online version contains supplementary material available at 10.1186/s40658-021-00417-z.

## Introduction

The perfusion component of a ventilation–perfusion single-photon emission computed tomography (VP SPECT) study is typically undertaken on the same day as, and frequently immediately following the ventilation component. When using technetium-99m (^99m^Tc)-based radiopharmaceuticals in a same-day imaging strategy (generally recommended, especially to obtain rapid diagnosis of pulmonary embolism) [1], residual ventilation counts contribute to the acquired perfusion images and may obscure perfusion defects leading to misdiagnosis. While software can perform correction for residual ventilation counts in the final perfusion image, slight differences in radiopharmaceutical distributions, and imperfect co-registration can lead to significant artefacts, and it is therefore recommended that the perfusion count rate (PCR—corrected for residual ventilation counts) exceeds the ventilation count rate (VCR) by a factor of at least 3 [2–4]. This is the equivalent, when a perfusion SPECT is performed immediately after the ventilation SPECT, of the uncorrected count rate exceeding the VCR by a minimum factor of 4. At the same time, higher ratios may also be considered undesirable since these would require high doses of the perfusion radiopharmaceutical [^99m^Tc]Tc—macroaggregated albumin ([^99m^Tc]Tc-MAA), which would result in higher radiation dose, and increased risks posed by the administration of a higher number of [^99m^Tc]Tc-MAA particles. The former is especially relevant in VP SPECT studies performed to diagnose pulmonary embolism (PE) in pregnant or lactating women in whom radiation exposure poses an additional risk; while the latter is relevant especially when administering [^99m^Tc]Tc-MAA in patients with known or occult disorders of the pulmonary vasculature.

The targeting of an optimal perfusion/ventilation count rate ratio (*P*/*V* ratio) is complicated principally by the fact that it is often difficult to quantify the amount of ventilation activity that has been administered in any individual patient. In patients with a large amount of soft tissue, higher amounts of administered activity may be required to obtain a sufficiently high VCR to guarantee a ventilation SPECT of sufficient quality. This is mainly because as the amount of soft tissue increases, there is increased attenuation due to Compton scatter [5]. Post hoc calculation of administered ventilation activity using knowledge of the imaging system sensitivity alone is not possible due to the frequently significant amount of attenuation by thoracic tissue. While absolute quantification of administered ventilation activity is possible using quantitative SPECT, this remains experimental and requires specialised software and a SPECT/CT system [6]. As a result, the dose and timing of [^99m^Tc]Tc-MAA administration after the ventilation SPECT is frequently suboptimal, resulting in PCRs that are either too low to achieve an adequate *P*/*V* ratio, or the administration of activities exceeding those recommended or required, and thus not meeting the principle of “as low as reasonably achievable” (ALARA).

It is possible to account for photon attenuation using information from a second, simultaneously acquired Compton scatter window [7]. Using various techniques in phantom-based studies, researchers have demonstrated linear relationships between the number of photons detected in scatter windows and voxel attenuation coefficients [8] or attenuation depth [9].

This study aimed to investigate whether a simple ratio of counts acquired in two adjacent energy windows on the ventilation SPECT (*E*_ratio_) is predictive of the final perfusion count rate normalised for [^99m^Tc]Tc-MAA activity (PCR_norm_) on the perfusion SPECT; and if so, whether such a relationship can be used to develop a simple tool to adjust either the *activity* (dose) of [^99m^Tc]Tc-MAA administered or the *timing* (or both) of the perfusion SPECT, such that an optimal *P*/*V* ratio may be achieved.

## Material and methods

### Participant selection

All patients referred to the Tygerberg Hospital Division of Nuclear Medicine for VP SPECT studies between July 2017 and July 2019 were eligible for inclusion. Only patients with same-day studies (ventilation followed by perfusion) and who were older than 18 years were included in this retrospective analysis. Patients in whom no [^99m^Tc]Tc-MAA injection site imaging was performed; or for whom the [^99m^Tc]Tc-MAA dispensing time was not recorded (to correct [^99m^Tc]Tc-MAA dose for residual counts at administration site, and for decay) were excluded, as were the scans of patients in whom the imaging acquisition differed from the divisional protocol; or in cases in which a repeat SPECT acquisition was performed. A second sample (*validation dataset*), subsequently drawn from scans performed between July 2019 and March 2020, with the same inclusion and exclusion criteria, was prepared to validate the tool developed with the original sample (*training dataset*).

### Imaging procedure

Ventilation was performed with the patient lying supine, using a [^99m^Tc]Tc-Technegas generator (Vita Medical Ltd, Lucas Heights, New South Wales, Australia). After each breath, tubing of the ventilation system was set aside and a Geiger-Müller (GM) detector (Radiation Alert® – Inspector, SE International Inc, Summertown, TN, USA) positioned on the sternum. Technicians were instructed to target an approximate stabilised probe count rate of ~ 2500 counts per minute (cpm). Immediately after ventilation, SPECT was performed on a dual-head Siemens Symbia SPECT camera (Siemens Healthineers, Hoffman Estates, IL, USA) with measured detector energy resolution of 8.5%, or a dual-head GE Infinia Hawkeye SPECT-CT camera (General Electric Medical Systems, Milwaukee, WI, USA) with measured detector energy resolution of 11.1%. Acquisition protocols (Table 1) were chosen to match most closely those recommended by Palmer et al. [4]. All SPECT scans were acquired with a primary energy window centred on a peak of 140 keV (width ± 7.5%); and a symmetrical down-scatter window, centred on a peak of 119 keV, of exactly the same width immediately beneath this. The latter window has been routinely included in VP SPECT acquisitions in the Tygerberg Division of Nuclear Medicine since 2015, at which time image reconstruction software (Hermes Gold 3; Nuclear Diagnostics, Stockholm Sweden) used this data to construct a synthetic attenuation map.

**Table 1:** SPECT acquisition parameters

Camera model	Siemens Symbia—dual detector	Infinia Hawkeye—dual detector
Collimators	LEAP	LEAP
Collimator sensitivity (measured) (cps/MBq)	133	119
Matrix	64 × 64	64 × 64
Zoom	1.45	1.3
Pixel size (mm)	6.8	6.8
Primary energy window centre (keV)	140	140
Primary energy window lower threshold (keV)	129.5	129.5
Primary energy window upper threshold (keV)	150.5	151
Secondary energy window centre (keV)	119	119
Secondary energy window lower threshold (keV)	108.5	108.5
Secondary energy window upper threshold (keV)	129.5	129.5
Frames	120	120
Angle step (degrees)	3	3
Rotation (degrees)	360	360
Frame duration ventilation SPECT (sec)	10	12
Frame duration perfusion SPECT (sec)	5	5

LEAP, Low-energy, all purpose; keV, kiloelectron volts; sec, seconds

The perfusion study was acquired either immediately after the ventilation study or after a delay to allow for some decay of ventilation counts using ~ 120 MBq of [^99m^Tc]Tc-MAA (Pulmocis®, Cisbio). All dispensed [^99m^Tc]Tc-MAA doses had a radiochemical purity of ≥ 95%. Static acquisitions (64 × 64 matrix, 60 s, 129.5–150.5 keV), using the same LEAP collimator as used for SPECT, were made of the [^99m^Tc]Tc-MAA injection site to exclude major misinjection and to quantify residual activity in the injection portal. Residual activity in the syringe was not measured.

### Data extracted from DICOM files and paper records

From the radiopharmacy and patient scan records, the time [^99m^Tc]Tc-MAA was dispensed and the activity at dispense time were recorded.

All raw tomographic VP SPECT image data, as well as corresponding injection site images for the periods in question were downloaded from the divisional PACS in DICOM format.

A custom script written in *Python* version 3.8.2 [10] using the *pydicom* package version 1.4.2 [11] was used to extract information from the DICOM image and header tags, both to assure compliance with divisional acquisition protocols, and to obtain the necessary image measures for analysis purposes.

Information to ensure consistency of the acquisition protocols included width and centring of the energy windows; scan arc; number of frames; frame duration; and camera and collimator identifiers. Scan order (ventilation followed by perfusion) was also validated by checking scan dates and times.

Measures extracted for analysis purposes included patient name, surname, and folder number (subsequently deidentified); patient gender and date of birth (for demographic purposes); scan dates and times (for decay correction); and total image counts for both energy windows (for tomographic data) or in the primary energy window for the injection site images.

Derived image measures and how they were calculated are summarised in Table 2.

**Table 2:** Derived image measures

Measure (unit)	Formula	Description
*E*_*ratio*_ (arb. unit)*	$E_{\mathrm {ratio}} = {{\sum \nolimits_{{i = 1}}^{{120}} v1_{i} } \mathord{\left/ {\vphantom {{\sum \nolimits_{{i = 1}}^{{120}} v1_{i} } {\sum \nolimits_{{i = 1}}^{{120}} v2_{i} }}} \right. \kern-\nulldelimiterspace} {\sum \nolimits_{{i = 1}}^{{120}} v2_{i} }}$	*E*_ratio_ was calculated by summing the counts of all projections in the primary energy window of the raw ventilation SPECT data ($v1$), and then dividing the result by the summed counts of all projections in the Compton scatter energy window of the raw ventilation SPECT data ($v2$)
VCR (cps)	${\text{VCR}} = {{\sum\nolimits_{{i = 1}}^{{120}} {v1_{i} } } \mathord{\left/ {\vphantom {{\sum\nolimits_{{i = 1}}^{{120}} {v1_{i} } } {t1}}} \right. \kern-\nulldelimiterspace} {t1}}$	The ventilation count rate (VCR) was calculated by summing the counts of all projections in the primary energy window of the raw ventilation SPECT data ($v1$), and then dividing the result by the ventilation SPECT acquisition duration ($t1$)
PVCR (cps)	${\text{PVCR}} = {{\sum\nolimits_{{i = 1}}^{{120}} {p1_{i} } } \mathord{\left/ {\vphantom {{\sum\nolimits_{{i = 1}}^{{120}} {p1_{i} } } {t2}}} \right. \kern-\nulldelimiterspace} {t2}}$	The perfusion + ventilation count rate (PVCR) was calculated by summing the counts of all projections in the primary energy window of the raw perfusion SPECT data ($p1$), and then dividing the result by the perfusion SPECT acquisition duration ($t2$)
PCR (cps)	${\text{PCR}} = {\text{PVCR}} - {\text{DF}} \cdot {\text{VCR}}$	The perfusion count rate (PCR) was calculated by first correcting *VCR* by the relevant decay factor [${\mathrm{DF}}$] to account for the interval between ventilation and perfusion SPECTs, and then subtracting the result from PVCR
*P/V* ratio (arb. unit)*	$\frac{P}{V}=\frac{{\mathrm{PCR}}}{{\mathrm{VCR}}}$	The *P/V ratio* was calculated by dividing PCR by VCR
*A*_inj_ (MBq)	$A_{{{\text{inj}}}} = {\text{DF}} \cdot {\text{ICR}}/S$	The activity of [^99m^Tc]Tc-MAA present at the injection site at time of P SPECT (*A*_inj_) was calculated by taking the count rate on the injection site planar static image (ICR), correcting this value by the relevant decay factor [${\mathrm{DF}}$] to account for the interval between perfusion SPECT and injection site imaging, and then dividing the result by camera-collimator sensitivity ($S$)
*A*_sys_ (MBq)	$A_{{{\text{sys}}}} = {\text{DF}} \cdot A_{{{\text{disp}}}} - A_{{{\text{inj}}}}$	The activity of [^99m^Tc]Tc-MAA administered systemically at the time of the perfusion SPECT (*A*_sys_) was calculated by first correcting the amount of activity at time of dispensing (*A*_disp_) by the relevant decay factor [DF] to account for the interval between dispensing and perfusion SPECT, and then subtracting *A*_inj_
PCR_norm_ (arb. unit)*	${p}_{\mathrm{rate}}=\frac{{\mathrm{PCR}}}{{A}_{{\mathrm{sys}}}}/S$	The normalised perfusion count rate (PCR_norm_) was calculated by dividing PCR by *A*_sys_, and then dividing the result by the camera-collimator sensitivity (*S*)

^*^Reported in Results section

### Modelling

Based on visual analysis of the initial scatter plot of the training dataset, a linear regression model of *E*_ratio_ and PCR_norm_ was built in *RStudio* version 1.3.1093 [12] using *R* version 4.0.2 [13]. Diagnostic plots were generated using native, *ggfortify* [14], and *nortest* packages. A threshold of *p* = 0.05 was considered statistically significant. Calculation of 95% prediction intervals for graphing purposes was performed using the native *predict.lm* function. Normality of the residuals was assessed using graphical methods.

### Prediction tool

Using the results of the linear regression performed on the training dataset, and the mean squared error (MSE), a tool to predict final *P*/*V* ratio was developed in Microsoft® Excel® for Microsoft 365 MSO (16.0.13127.20620) 64-bit.

### Validation

Validation of the Excel-based tool was performed by comparing the model-predicted *P*/*V* ratio values with the actual *P*/*V* ratios for studies in the validation dataset. Accuracy of the model in predicting *P*/*V* ratio was assessed by calculating the mean absolute percentage error (MAPE) using the *MLmetrics* package in *R*.

### Clinical impact

The practical implications of using the tool on studies included in the validation dataset was also simulated for a sample strategy that favoured dose sparing. First, VCR values were normalised such that the summed ventilation counts equalled the recommended value of 2 million [4]. Because the actual PCR per MBq of administered [^99m^Tc]Tc-MAA could be retrospectively calculated, it was possible to (1) calculate the [^99m^Tc]Tc-MAA dose that would have been required to achieve a minimum *P*/*V* ratio of 3 had the perfusion component immediately followed the ventilation component (i.e. no delay), and (2) to calculate the true delay that would have been required after an optimal ventilation study before it would have been possible to proceed with a target dose (120 MBq) of [^99m^Tc]Tc-MAA and still achieve an adequate *P*/*V* ratio. These results were then compared to the delay recommended by the prediction tool, assuming a [^99m^Tc]Tc-MAA dose of 120 MBq and the same minimum *P*/*V* ratio. The 120 MBq target dose is the same as that recommended in a same-day protocol in pregnant patients [15] and provides a margin of safety should the actual dose required while injecting under the detector be greater (up to the limit of 185 MBq according to the package insert for the Pulmocis® product). By comparing the *true delay* required and the *predicted delay* required, *unnecessary delay* (delay suggested by model exceeding actual delay required) and *insufficient delay* (delay suggested by model inadequate) could also be quantified. Benefits of using the prediction tool were quantified in terms of dose of [^99m^Tc]Tc-MAA spared. Costs of using the prediction tool were quantified in terms of unnecessary delay. Since the demonstration of clinical impact was performed purely for illustrative purposes using one of many possible strategies, confidence intervals for the predicted delays required were ignored for the sake of simplicity.

## Results

### Selection of participant scans

#### Training dataset

A total of 643 studies (ventilation SPECT followed by same-day perfusion SPECT) were identified in the study period. Of these, 22 were of patients under the age of 18 years and were excluded. An additional 199 studies were excluded due to missing injection site image; one significant misinjection (83 MBq); 69 due to missing radiopharmacy records ([^99m^Tc]Tc-MAA dispense time not recorded); four due to incorrect dispense time records (recorded time later than scan time); and one due to deviation from the divisional protocol (incorrect collimator used). Four participants had repeat studies (10.1–58.6 weeks later), and for these participants, only the first study was included in the analysis. An initial analysis was performed on 343 imaging studies. One imaging study was retrospectively excluded after it became apparent that it was an outlier with high influence and an obvious cause was identified (details below). The sample size for the final analysis of the training dataset was thus of 342 imaging studies.

### Validation dataset

A total of 285 additional same-day studies were identified in the second period for use in the validation dataset. Of these, six were of patients under the age of 18 years and were excluded. An additional 71 studies were excluded due to missing radiopharmacy records ([^99m^Tc]Tc-MAA dispense time not recorded); eight due to incorrect dispense time records (recorded time later than scan time); one significant misinjection (36 MBq); and one due to a missing injection site image. No participants in this sample had repeat studies. The sample size for the validation dataset was thus 198 studies.

### Demographics and summary statistics

Of the 342 imaging studies included in the final analysis of the training dataset, 317 were performed in female patients. The median patient age was 30.1 years (IQR: 25–37.2 years). Mean *A*_sys_ was 114.8 MBq (SD: 8 MBq). Mean *E*_ratio_ was 1.83 (SD: 0.21), and mean PCR_norm_ was 0.313 (SD 0.067). Mean *P*/*V* ratio was 4.01 (SD 1.09).

Of the 198 imaging studies included in the final analysis of the validation dataset, 188 were performed in female patients. The median patient age was 29.7 years (IQR: 24.6–37.5 years). Mean *A*_sys_ was 118.4 MBq (SD: 9.9 MBq). Mean *E*_ratio_ was 1.8 (SD: 0.23), and mean PCR_norm_ was 0.311 (SD 0.072). Mean *P*/*V* ratio was 4.98 (SD 1.44).

A breakdown of the final *P*/*V* ratio values in the training and validation datasets appears in Table 3.

**Table 3:** * P*/*V* ratio values of the studies included in the training and validation datasets

*P*/*V* ratio	Training dataset	Validation dataset
	Number of studies (%)	Number of studies (%)
1–2	7 (2%)	0 (0%)
2–3	52 (16%)	4 (2%)
3–4	114 (34%)	57 (29%)
4–5	105 (31%)	50 (25%)
> 5	56 (17%)	87 (44%)
Total	343 (100%)	198 (100%)

### Linear regression

A simple linear regression of the training data was calculated to predict PCR_norm_, given *E*_ratio_. The initial scatter plot and diagnostic charts (Additional file 1: Fig. S1) identified an obvious outlier (datapoint 223: *x* = 1.89, *y* = 0.62). Further investigation revealed that the ventilation SPECT in this case had a very high amount of activity outside of the patient which very likely influenced the *E*_ratio_ value (Additional file 2: Fig. S2). Given that this observation was both an outlier and highly influential, and because an obvious cause was identified on review of the raw data, this observation was retrospectively excluded.

After exclusion of the outlier, a significant regression equation was found (*F*(1,340) = 1057.3, *p* < 0.0001), with an adjusted *R*^2^ of 0.756 and MSE of 0.001089. After adjusting for any specific camera-collimator sensitivity factor, the relationship to yield a re-normalised (adjusted) perfusion count rate was represented by the following equation:

$${\text{PCR}}\, = \,A \times S \times \left( {0.28\left( {E_{{{\text{ratio}}}} } \right) - 0.198} \right)$$

where PCR represents the expected perfusion count rate (in cps), for any administered activity (*A*) of [^99m^Tc]Tc-MAA (in MBq), re-normalised (adjusted) to account for the user-specific sensitivity of the camera-collimator combination $S$, and predicted by *E*_ratio_.

**Fig. 1:**
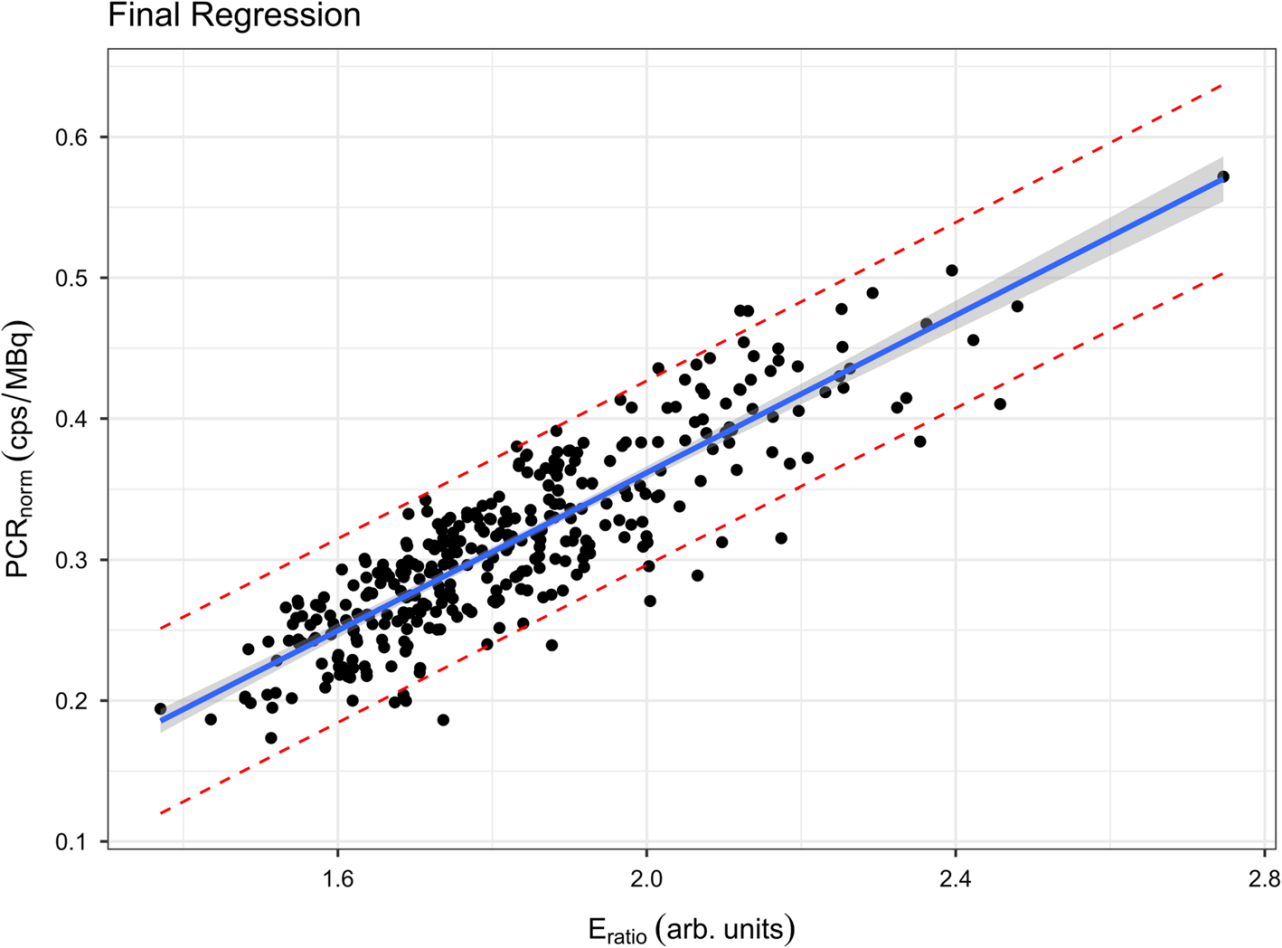
Final linear regression of perfusion count rate normalised to 1 MBq of administered [^99m^Tc]Tc-MAA, and a camera-collimator sensitivity of 1 cps/MBq (PCR_norm_) as a function of the dual energy window count ratio of the ventilation study (*E*_ratio_). Regression line (blue solid line), 95% confidence band (dark grey), and 95% prediction bands (red dashed lines). Image generated in *RStudio*

The final model, including confidence and prediction intervals is plotted in Fig. 1. The relevant diagnostic plots for the final regression appear in Additional file 3: Fig. S3 and Additional file 4: Fig. S4.

### Prediction tool

The output of the linear regression enables calculation of the prediction interval around *y*, for any future value of *x* using the equation:

$$\hat{y} \pm t_{{(\frac{\alpha }{2},n - 2)}} \times \sqrt {{\text{MSE}} \times \left( {1 + \frac{1}{n} + { }\frac{{\left( {x_{h} - \overline{x}} \right)^{2} }}{{\sum \left( {x_{i} - \overline{x}} \right)^{2} }}} \right){ }}$$

where $\hat{y}$ is the predicted fitted value of $y$ for a particular value of $x$; ${t}_{(\frac{\alpha }{2},n-2)}$ represents the two-tailed inverse of the Student's t distribution with significance level $\alpha$ for $n$ number of observations in the regression dataset; MSE is the mean squared error; ${x}_{h}$ is the value of a hypothetical future observation of $x$; ${\bar{x}}$ is the mean value of $x$ in the regression dataset; and ${x}_{i}$ represents the respective values of $x$ in the regression dataset [16].

Using the above formula, a practical tool to predict the final *P*/*V* ratio (with 90% and 95% prediction intervals) was developed in Excel (Additional file 6). A video demonstrating practical implementation of the tool is included in Additional file 7.

### Validation

**Fig. 2:**
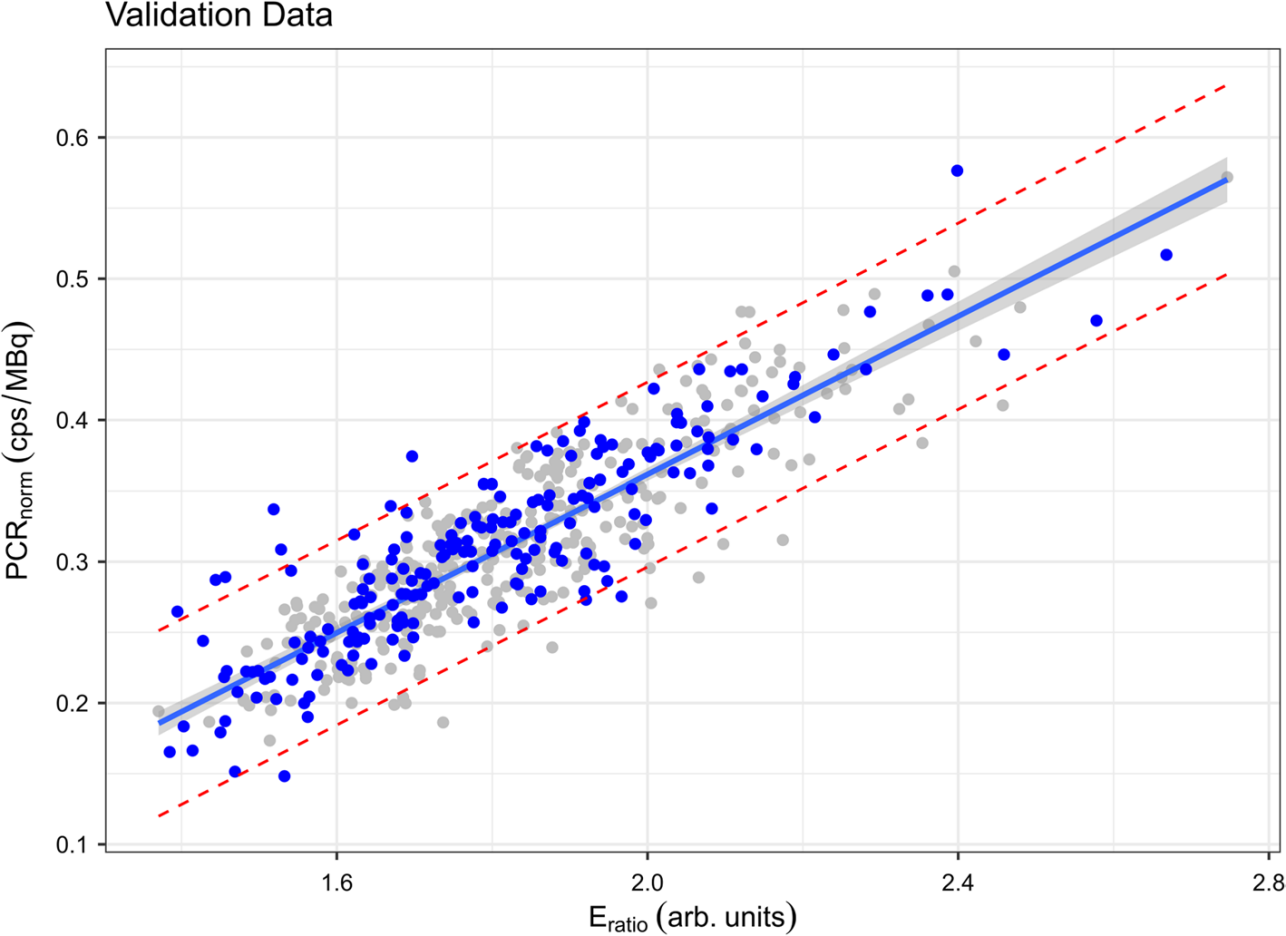
Final linear regression as shown in Fig. 1, with training dataset values in grey and superimposed validation dataset values in blue. Image generated in *RStudio*

**Fig. 3:**
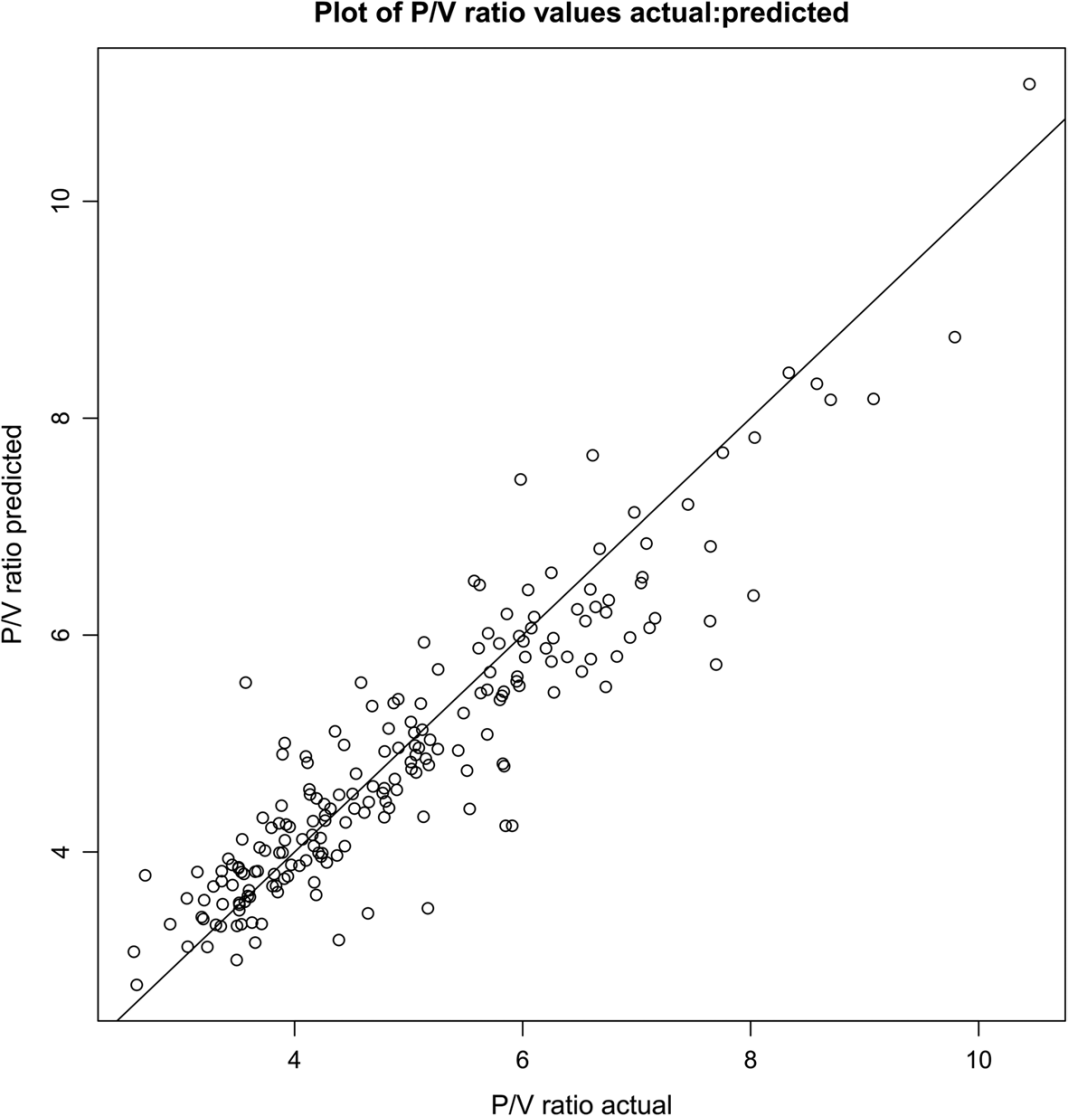
Scatter plot of *P*/*V* ratio values predicted with the Excel-based tool versus actual values (validation dataset). The straight line indicates points for which *P*/*V* ratio predicted = *P*/*V* ratio actual. Image generated in *RStudio*

A scatter plot of predicted *P*/*V* ratios for the validation dataset overlayed on the linear model developed using the training dataset is shown in Fig. 2. A plot comparing actual *P*/*V* ratios to predicted values appears in Fig. 3. The prediction tool was 91% accurate in predicting *P*/*V* ratios in the validation dataset (MAPE of 9%).

### Clinical impact

In the simulation using data from the validation dataset, had the [^99m^Tc]Tc-MAA dose been injected blindly (without using the prediction tool) immediately after an optimal ventilation study, the required activity would have exceeded the target dose of 120 MBq in 56/198 cases, of which seven would have exceeded the maximum recommended dose of 185 MBq.

**Fig. 4:**
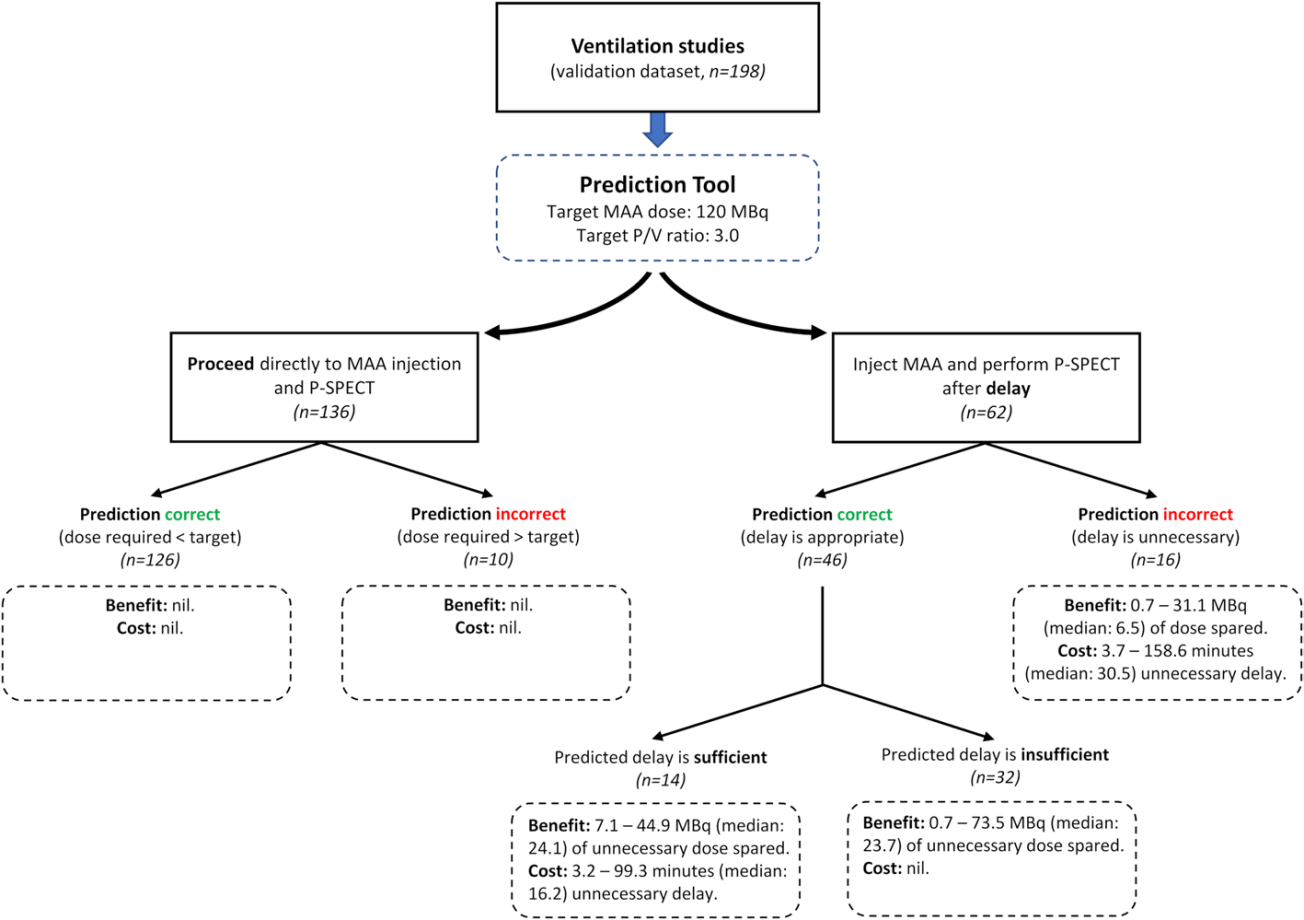
Results of applying the prediction tool to ventilation studies in the validation dataset using a dose-sparing strategy (targeting a [^99m^Tc]Tc-MAA dose of 120 MBq, and a *P*/*V* ratio of 3). In the stimulation, values predicted by the tool were compared to equivalent values calculated directly from actual perfusion data

A breakdown of the impact of using the prediction tool is shown in Fig. 4. Overall, use of the prediction tool would have delayed the perfusion study in 62 patients resulting in a dose sparing of 0.7–73.5 (median 19.7) MBq at a cost of 3.2–158.6 (median 20.2) minutes. Following the recommendation of the prediction tool would have resulted in 14 additional patients requiring a [^99m^Tc]Tc-MAA dose less than 120 MBq and six patients being spared excessive doses above 185 MBq.

## Discussion

This study found that the relationship between the ratio of ventilation counts in the primary energy window and an adjacent Compton scatter window and the final perfusion count rate after the administration of a given dose of [^99m^Tc]Tc-MAA could be linearly approximated. Results of the analysis were used to develop a practical, Excel-based tool to predict the adequacy of the *P*/*V* ratio in same-day VP SPECT studies, which nuclear medicine practitioners can use to optimise the trade-offs that must frequently be made in these studies. This tool was validated using a second, training dataset and found to be accurate in predicting final *P*/*V* ratio. The clinical impact of using the tool was illustrated using a dose-sparing strategy.

In a same-day VP SPECT study, the ventilation component is followed (ideally immediately after) by the perfusion component. Different strategies are used to increase the likelihood of obtaining ventilation images that are of sufficiently high diagnostic quality, while still meeting a final target *P*/*V* ratio of ≥ 3, and adhering to recommended [^99m^Tc]Tc-MAA doses. Most divisions target a specific count rate (e.g. 2000–2500 cpm) [17, 18] measured using a GM detector after ventilation, and having achieved this, proceed to ventilation imaging. Depending on the count rate on the final ventilation SPECT, centres either proceed immediately with injection of [^99m^Tc]Tc-MAA under the camera detector, usually checking for or titrating to a fourfold increase in count rate, or delay the perfusion component of the study until such a time that ventilation count rate is judged to have sufficiently decayed.

There are several problems with these strategies. Foremost, that the VCR is attenuated by variable amounts of soft tissue. This has the effect that a given activity (e.g. 40 MBq) of administered ventilation agent might result in a very high VCR (in a thin patient with minimal attenuating soft tissue), or a very low VCR (in a patient with more thoracic soft tissue), typically requiring the additional administration of ventilation agent. Very high count rates on the final ventilation study may lead to unnecessary deferment of the perfusion study in the first patient (in whom immediately proceeding with, e.g. a 120 MBq dose of [^99m^Tc]Tc-MAA might in actual fact have been sufficient), or lead to an inadequate *P*/*V* ratio in the second patient (who might require, e.g. 80 MBq of administered ventilation agent to achieve adequate ventilation counts) when their perfusion study is undertaken immediately, even with the maximum [^99m^Tc]Tc-MAA dose of 185 MBq recommended by the Pulmocis® package insert. Titrating the dose of [^99m^Tc]Tc-MAA administered to count rate under the camera is a useful compromise [19] but may require excessive amounts of activity (and particles) with attendant risks. It may be speculated that the difficulties in optimising the trade-offs between dose, count adequacy, sufficient *P*/*V* ratio, and reasonable patient turnaround times, are greatest in units caring for patients with wide variability in body habitus. Certainly, these trade-offs are frequently imperfect: a multicentre survey including the results of 286 VP SPECT studies in Germany reported inadequate *P*/*V* ratios in 25% of studies [19] which is similar to the findings of the current study, in which 18% of studies in the training dataset did not meet the recommended *P*/*V* ratio of > 3.

A *P*/*V* ratio that is too high is also problematic. While the optimal *P*/*V* ratio is uncertain, it seems unlikely that much additional diagnostic benefit is achieved with ratios above 5 (44% of cases in the validation dataset). While [^99m^Tc]Tc-MAA is generally accepted as a safe compound, it is not entirely innocuous, with reports of respiratory complications and death when high particle numbers have been administered to patients with underlying pathology of the pulmonary vasculature [20–23]. The risks posed by exposure to low doses of ionising radiation are more controversial [24, 25] but certainly it is reasonable to try and minimise the risks conferred by both particle number and radiation when reasonably achievable.

Most SPECT cameras can be easily configured to simultaneously image two adjacent energy windows. Once the ventilation SPECT is complete, it is a simple matter to perform a quick quality check of the raw data, and to use the perfusion adequacy prediction tool to predict the final *P*/*V* ratio. Moreover, this tool allows users to prioritise lower radiation dose or higher scan quality by modelling the impacts of different [^99m^Tc]Tc-MAA doses and/or delays before performing the perfusion SPECT on the *P*/*V* ratio.

Compton scatter is the predominant mode of gamma photon interaction with matter when imaging with [^99m^Tc]Tc-based tracers and increases as the depth of attenuating soft tissue increases [9]. This is evidenced by plotting *E*_ratio_ values against body mass index (a crude measure of body habitus) for a subset (*n* = 291) of participants from the training dataset in whom this information was available (Additional file 5: Fig. S5a). This also explains why *E*_ratio_ for any individual patient is constant, independent on the amount of administered activity (dose) since the attenuation profile remains constant—illustrated by plotting *E*_ratio_ values derived from the V SPECT study with *E*_ratio_ values derived from the corresponding VP SPECT for the combined dataset (Additional file 5: Fig. S5b).

A linear model to describe the relationship between *E*_ratio_ and PCR_norm_ was selected based on the scatter plot of data in the training dataset but the basis for such a relationship is uncertain. Researchers have previously demonstrated an approximately linear relationship between scatter fraction and attenuation: Sitek et al. demonstrated a linear relationship between the proportion of singly scattered photons and voxel attenuation coefficients, which they used to calculate mu-maps in a [^99m^Tc]Tc torso phantom study [8]. Similarly, also using phantoms, another group showed that ratios of scatter photons to photons detected in the primary energy window can be linearly correlated with tissue depth [9]. These linear relationships, assuming they translate to real world conditions, would however still not explain a linear relationship between *E*_ratio_ (calculated on the ventilation study raw data) and PCR in the current study, since the latter would still be subject to the phenomenon of exponential attenuation of photon transmission in broad beam conditions. Rather, it is speculated that the relationship is in fact exponential, and that the success of the linear model in this study is because a linear approximation is sufficiently accurate over the range of tissue depths encountered in the sample (not quantified in this experiment).

In addition to enabling the validation of the model, the second dataset allowed a simulation of clinical impact when using the prediction tool. For the purpose of illustration, a dose-sparing strategy was chosen, mimicking the preferred practice of the Tygerberg Hospital Division of Nuclear Medicine. It is however important to emphasise that the trade-off between dose and delay can be set to any user’s institutional or personal preference when using the tool. Using a dose-sparing strategy in this simulation, compared to values derived from the actual measures, the Excel-based tool would have appropriately deferred the majority of perfusion studies that would otherwise have required activities of [^99m^Tc]Tc-MAA exceeding the target dose of 120 MBq, including those exceeding the 185 MBq maximum limit recommended by the manufacturer, and this would have been achieved with only the minimal cost of excessive delay in a few cases.

This study suffered from several limitations. Many scans had to be excluded from the analysis due to missing injection site imaging or radiopharmacy records, which affected the final sample size. The sample was mostly composed of young (adult), pregnant women which means that caution should be exercised when applying the results to other patient demographics, although no reason for deviation from the model is expected on in other patient groups. More importantly, the linear relationship between *E*_ratio_ and PCR_norm_ at extreme values of *E*_ratio_ was based on only a few observations, and the validity of the linear model at these extremes is less certain. Confidence and prediction intervals may have been narrower had this study included additional corrections potentially affecting the accurate calculation of the amount of systemically administered [^99m^Tc]Tc-MAA(*A*_sys_): (a) for residual activity in the syringe after administration, and (b) for the interval between injection and the perfusion SPECT. The latter limitation is however not expected to be a major contributor to error, given the divisional policy of commencing the perfusion SPECT immediately post-injection. Additional sources of error not accounted for include non-correction for out-of-field-of-view scatter, and variation in the radiochemical purity of [^99m^Tc]Tc-MAA between 95 and 100% (which may have contributed slightly to error in PCR_norm_). Another, inherent limitation in predicting PCR is presented by variability in right–left shunting (including physiological shunting) which could not be accounted or corrected for [26]. Finally, it is also important to acknowledge that these results are specific for the energy window settings used on systems with an ~ 10% energy resolution, and using [^99m^Tc]Tc-Technegas, which has minimal early clearance after ventilation [27]. Is not clear how changes in these acquisition setting or of the ventilation agent used, might affect the reliability of the model.

## Conclusion

The expected perfusion count rate for a given dose of [^99m^Tc]Tc-MAA can be linearly predicted by the ratio of ventilation counts in two adjacent energy windows. This relationship allows for the application of a simple tool to predict and/or optimise the final *P*/*V* ratio. In addition to the practice of directly monitoring count rate increases during [^99m^Tc]Tc-MAA injection, this tool may assist in optimising the trade-offs between study quality, [^99m^Tc]Tc-MAA dose, and patient turnaround time while still achieving diagnostic quality VP SPECT studies.

## Supplementary Information

**Additional file 1: Fig 1** Diagnostic plots of initial training dataset. a Scatter plot of observations in which Eratio represents the calculated ratio between acquired counts in the primary energy window divided by the down-scatter window on the ventilation study and PCRnorm represents the perfusion count rate per megabecquerel of administered [99mTc]Tc-MAA (cps/MBq), normalised to a camera-collimator sensitivity of 1 cps/MBq. b Chart of standardised residuals identified an obvious outlier (observation 223), which was also shown to be highly influential on c Cooks’ distance chart. Investigation of this observation identified an obvious cause (Figure 2), and on this basis the case was excluded, and the regression repeated. Images generated in RStudio.

**Additional file 2: Fig 2** Reconstructed ventilation SPECT of participant 223 (anterior and lateral projections), exhibiting high amount of extracorporeal radiopharmaceutical (red crosshair), very likely on the basis of collimator/detector contamination, resulting in an unreliable Eratio value. This participant was excluded from the final analysis. Image generated in Hermes Hybrid Viewer PDR 4.0.1 with linear BW inverse colour table, relative scaling.

**Additional file 3: Fig 3** Diagnostic plots of final training dataset, exhibiting a linearity of the data, b normality of the residuals, c homogeneity of variance, and d outlier observations and points with high leverage. Abbreviations: lm: linear model; PCRnorm: normalised perfusion count rate; Eratio: energy window count ratio of the ventilation study. Images generated in RStudio.

**Additional file 4: Fig 4** Q-Q (top row) and density (bottom row) plots of the residuals (linear regression of PCRnorm on Eratio), for the training (left), validation (middle) and combined (training+validation, right) datasets. Results illustrate the close adherence to normal distribution of the residuals and support the use of parametric methods to build a prediction tool using the training dataset. Images generated in RStudio.

**Additional file 5: Fig 5** Scatter plots illustrating the relationship between body habitus (body mass index—BMI) and Eratio (a), as well as that Eratio values for any individual subject on V SPECT and VP SPECT are equal (R2 = 0.98) (b). The straight line indicates points for which Eratio values calculated on the two studies are equal. Images generated in RStudio.

**Additional file 6**. Perfusion adequacy prediction tool.

**Additional file 7**. Explanatory video.

## Data Availability

Regression data are included in additional files. Other non-identifying data are available from the corresponding author on reasonable request.

## Code availability

*Python* and *R* scripts are available from the corresponding author on reasonable request.
