# Supplementary figures and images for: Dual energy window imaging for optimisation of P/V ratios in VP SPECT
Source: EJNMMI Phys. 2021 Oct 16;8:69. doi: 10.1186/s40658-021-00417-z (PMC8520548; doi:10.1186/s40658-021-00417-z)

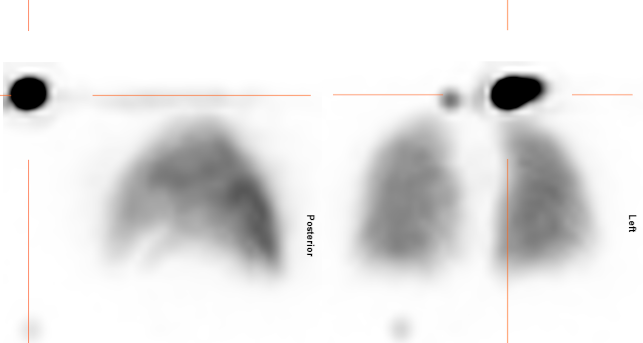

Supplement: Supplementary file 2 — Additional file 2: Fig 2 Reconstructed ventilation SPECT of participant 223 (anterior and lateral projections), exhibiting high amount of extracorporeal radiopharmaceutical (red crosshair), very likely on the basis of collimator/detector contamination, resulting in an unreliable Eratio value. This participant was excluded from the final analysis. Image generated in Hermes Hybrid Viewer PDR 4.0.1 with linear BW inverse colour table, relative scaling. [file 40658_2021_417_MOESM2_ESM.tiff]
